# “Care is needed the most, when it is deserved the least” – the experience of BPD-women

**DOI:** 10.1192/j.eurpsy.2022.1583

**Published:** 2022-09-01

**Authors:** M.E. Jensen, K. Andreasson, M. Vinberg, L. Oestergaard

**Affiliations:** 1 Mental Health Services in the Capital Region of Denmark, Mental Health Centre North Zealand, Hilleroed, Denmark; 2 Odense University Hospital, Department Of Urology, Odense, Denmark

**Keywords:** Qualitative research, stigmatization, borderline personality disorder, BPD

## Abstract

**Introduction:**

BPD are often characterized by dependence, affectability, unpredictability, impulsivity and self-destructiveness. Paradoxically, the symptoms associated with BPD are the same behaviors that makes them difficult to accommodate by health professionals. They constitute the most excluded and stigmatized patient group.

**Objectives:**

To gain knowledge on how BPD patients felt acknowledged when they experienced the need for professional help.

**Methods:**

We conducted semi-structured interviews with six BPD-women, aged between 18 to 46, all inpatient at different psychiatric units in the Capital Region of Denmark. The data were analyzed and interpreted through meaning condensation. We entered the philosophical hermeneutic framework of Hans-Georg Gadamer.

**Results:**

We found that the women experienced that; the diagnosis was a filter, in which they were always viewed and judged through as “just another BPD-patient” and not a unique individual. their cry for help was expected to be verbalized in a certain manner and therefore was often not understood nor heard, but instead they experienced to be scolded by health professionels. the emergency plan became a legitimate way for the health care professionals to avoid spending to many resources, rather than a helpful tool. the psychiatry as a unit was largely characterized by stigmatization and a distrustful attitude towards them. Therefore they felt deeply dependent on meeting that one special health professional who were experienced to have a genuine interest and desire to help them.

**Conclusions:**

Findings correspond with the findings of existing research. Hence, there also seems to be significant barriers nationally for patients with BPD to experience being acknowledged and helped, when in need of professional help.

**Disclosure:**

No significant relationships.

